# Abdominal Wall Endometriosis Following Cesarean Section: A Retrospective Analysis

**DOI:** 10.3390/jcm15145615

**Published:** 2026-07-17

**Authors:** Süreyya Sarıdaş Demir, Serem Kel Ilgın, Mehmet Nuri Duran, Özgür Şahin, Bülent Demir, Nihal Kılınç

**Affiliations:** 1 Department of Perinatology, Private Clinic, Çanakkale 17000, Türkiye; 2 Department of Obstetrics and Gynecology, Biga State Hospital, Çanakkale 17000, Türkiye; 3 Department of Obstetrics and Gynecology, Ezine State Hospital, Çanakkale 17000, Türkiye; 4 Department of Obstetrics and Gynecology, Faculty of Medicine, Çanakkale Onsekiz Mart University, Çanakkale 17000, Türkiye; 5 Department of Pathology, Faculty of Medicine, Çanakkale Onsekiz Mart University, Çanakkale 17000, Türkiye

**Keywords:** abdominal wall endometriosis, cesarean section, intramuscular endometriosis, scar endometriosis, rectus abdominis, wide local excision, anesthetic management, regional anesthesia, case series, Pfannenstiel incision

## Abstract

**Background**: Abdominal wall endometriosis (AWE) following cesarean section (CS) is an increasingly recognized complication. Intramuscular involvement of the rectus abdominis and the management of associated fascial defects remain underreported. **Methods**: We retrospectively analyzed 14 consecutive women with histopathologically confirmed AWE following CS. Clinical presentation, imaging, operative technique, and follow-up outcomes were analyzed. **Results**: Mean age was 35.4 years (range 29–45). Three cases (21.4%) showed intramuscular rectus abdominis invasion; 11 (78.6%) were subcutaneous. Mean nodule size was 24.1 mm (range 9–45 mm). Cross-sectional imaging (MRI or CT) was obtained selectively in four patients with the largest lesions. Wide local excision with free margins was achieved in all cases. Two intramuscular cases required primary fascial repair (defects ~2 cm) without prosthetic mesh. No recurrences were detected at 12–24 months of follow-up. **Conclusions**: AWE following CS may involve both subcutaneous and intramuscular planes. Wide local excision with primary fascial repair is effective for small defects. Preoperative sonographic evaluation and individualized operative planning are essential for optimal outcomes.

## 1. Introduction

Endometriosis is defined as the ectopic growth of endometrial glands and stroma outside the uterine cavity, affecting approximately 2–4% of women of reproductive age and associated with pelvic pain, dysmenorrhea, and infertility [1,2]. Among its extrapelvic manifestations, abdominal wall endometriosis (AWE) represents a clinically important but frequently underdiagnosed entity [3].

AWE most commonly arises as a complication of cesarean section (CS), with a reported incidence of 0.03–3.5% [4,5,6]. The prevailing pathogenetic mechanism is iatrogenic transplantation of endometrial cells into the abdominal wall wound at the time of surgery, followed by cyclical hormonal stimulation and proliferation [7]. As global CS rates continue to rise, the incidence of AWE is expected to increase accordingly [8,9].

The typical presentation is a painful, palpable mass at or near the Pfannenstiel incision scar, with pain that often follows a cyclic pattern correlated with menstruation [4,10]. Diagnosis is frequently delayed due to limited clinical awareness and a broad differential diagnosis including lipoma, hematoma, abscess, suture granuloma, incisional hernia, and malignancy [11]. Ultrasonography (US) is the recommended first-line imaging modality; magnetic resonance imaging (MRI) provides additional information for deep or atypical lesions [12,13].

Wide local excision (WLE) with adequate margins is the definitive treatment [14,15]. Intramuscular invasion of the rectus abdominis occurs in a subset of cases and carries specific surgical implications—including the need for muscle resection and fascial reconstruction—that are seldom systematically described in the literature.

Here we report a retrospective case series of women with AWE following CS, with specific emphasis on surgical management of intramuscular rectus abdominis involvement and primary fascial reconstruction without prosthetic mesh.

## 2. Materials and Methods

### 2.1. Study Design and Ethics

This retrospective case series was approved by the Non-Interventional Clinical Research Ethics Committee of Çanakkale Onsekiz Mart University (Approval No.: 2026-110; Date: 8 April 2026). The study was conducted in accordance with the Declaration of Helsinki.

### 2.2. Patient Selection

Inclusion criteria were: (1) history of at least one prior CS; (2) palpable abdominal wall mass at or near the CS scar; (3) surgical excision performed within our department; and (4) histopathological confirmation of endometriosis. Surgical intervention was indicated in all patients presenting with symptomatic abdominal wall masses and imaging findings consistent with AWE. The 14 patients included represent all consecutive cases diagnosed and surgically managed within our Obstetrics, Gynecology, and Perinatology department during the study period who fulfilled these criteria. The total number of AWE cases at our institution may be higher: some patients diagnosed at our center subsequently underwent surgery at other institutions and lacked histopathological documentation, precluding their inclusion. Cases managed by the General Surgery department were outside the scope of departmental records and were therefore not captured. Both sources of potential underascertainment are acknowledged in the Limitations section.

### 2.3. Preoperative Evaluation

All patients underwent clinical examination and abdominal wall ultrasonography (US). Lesion location was classified as subcutaneous (above the anterior rectus fascia) or intramuscular (involving the rectus abdominis muscle). In four patients, US was supplemented by cross-sectional imaging owing to equivocal depth of involvement or lesion size: MRI was performed in three patients (Cases 8, 13, and 14) and CT in one (Case 12) [12,13].

### 2.4. Surgical Technique

Wide local excision (WLE) was performed in all cases with a macroscopic margin of at least 1 cm of healthy surrounding tissue. Anesthetic management was individualized according to lesion depth and patient characteristics: two patients received combined local infiltration (0.5% bupivacaine with epinephrine) and spinal anesthesia; eleven were managed with spinal anesthesia alone; and one patient with a complex intramuscular lesion required general anesthesia.

For subcutaneous lesions, excision included the overlying skin, subcutaneous fat, and any involved anterior rectus fascia, with primary fascial repair as needed. For intramuscular lesions (Cases 6, 8, and 13), the affected rectus muscle fibers were excised en bloc with the nodule; the posterior rectus sheath and peritoneum were preserved in all cases. Primary muscular and fascial repair was performed with non-absorbable sutures. No prosthetic mesh was required in any case.

### 2.5. Follow-Up

Patients were reviewed clinically at 4–6 weeks and 12 months postoperatively. An abdominal wall US was obtained at 12 months to assess local recurrence.

## 3. Results

### 3.1. Patient Demographics

Fourteen women with a mean age of 35.4 ± 6.1 years (range 29–45) were included. The mean number of prior CSs was 1.8 (range 1–3); mean gravidity was 2.7 (range 1–5) and mean parity was 2.1 (range 1–3). All patients were of reproductive age and presented with a mass at the CS scar.

### 3.2. Clinical Presentation and Imaging

Cyclic pain concordant with menstruation was the predominant symptom pattern (10/14, 71.4%). Three patients (21.4%) had cyclic pain as the main complaint, four (28.6%) presented primarily with a palpable mass, and seven (50.0%) reported both. Preoperative US in all 14 patients demonstrated hypoechoic or heterogeneous semi-solid masses consistent with endometrioma.

### 3.3. Lesion Location and Size

Each patient presented with a single endometriotic nodule. Mean nodule size was 24.1 mm (range 9–45 mm). Three lesions (21.4%) involved the rectus abdominis muscle (intramuscular; Cases 6, 8, and 13); the remaining 11 (78.6%) were confined to the subcutaneous plane. Nodule location along the Pfannenstiel incision was right-sided in five cases (35.7%), left-sided in five (35.7%), and central in four (28.6%). Cross-sectional imaging was obtained in four patients: MRI in Cases 8, 13, and 14, and CT in Case 12. All demographic, clinical, and surgical data are summarized in Table 1.

### 3.4. Intraoperative Findings

**Figure 1:**
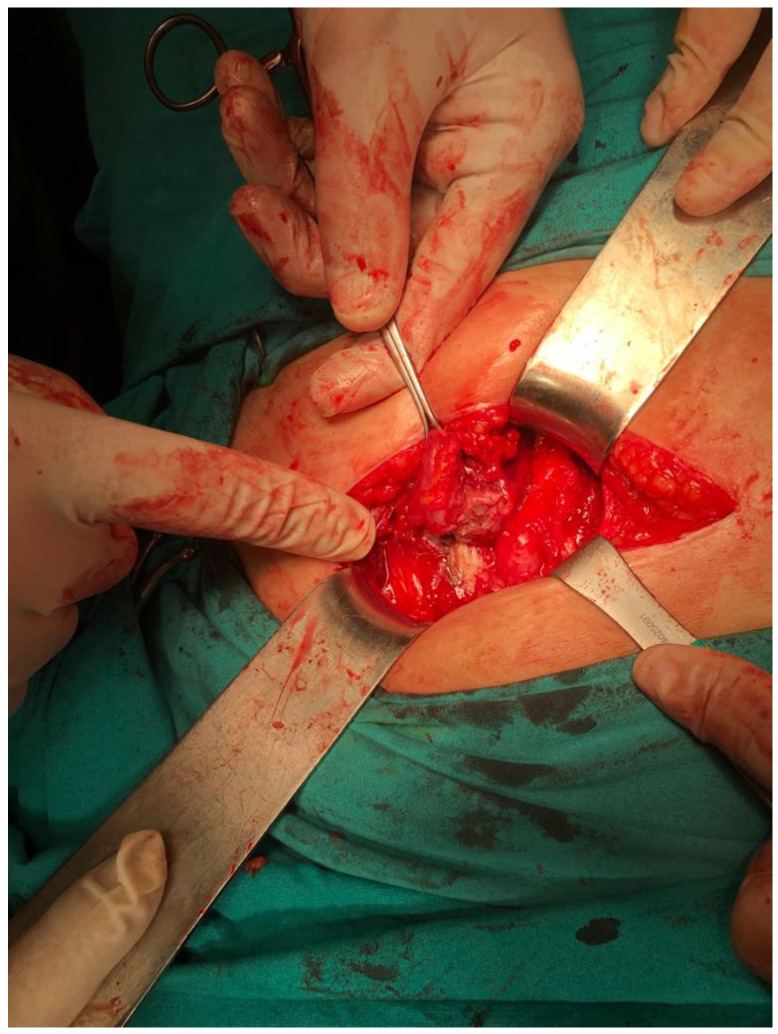
Intraoperative appearance during wide local excision of an intramuscular endometriotic nodule from the rectus abdominis muscle (Case 8). Retractors provide wide exposure of the operative field. The characteristic reddish-brown nodule is visible within the muscle belly; circumferential dissection with a ≥1 cm margin of healthy muscle tissue is demonstrated. The posterior rectus sheath (indicated by the surgeon’s finger) was preserved intact.

No intraoperative complications were recorded in any case. For intramuscular lesions, the nodule was excised en bloc with the affected muscle fibers. Two of the three intramuscular cases required fascial repair; defects measured approximately 2 cm and were closed primarily without mesh augmentation. Figure 1 illustrates the intraoperative appearance during excision of an intramuscular nodule.

**Figure 2:**
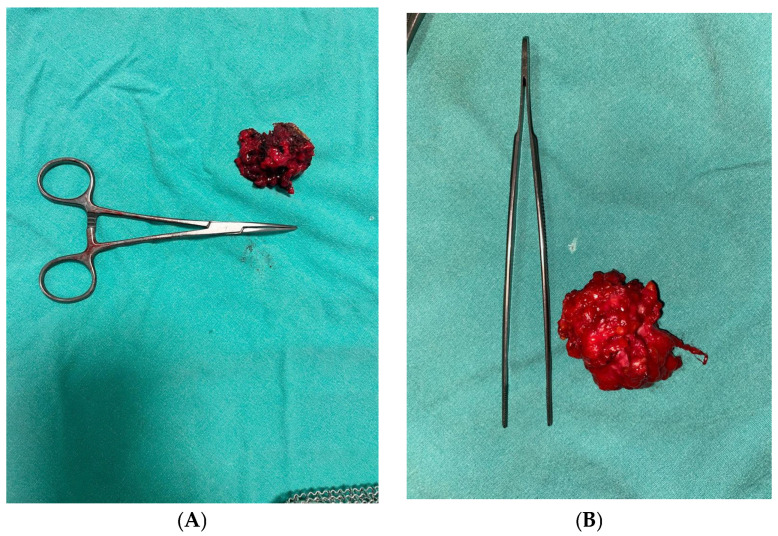
Macroscopic appearance of excised endometriotic specimens from two patients with intramuscular rectus abdominis endometriosis. (**A**) A well-circumscribed reddish-brown nodule measuring 28 mm is shown alongside a standard Kelly clamp for scale. (**B**) A second specimen measuring 35 mm is shown alongside surgical forceps for scale. Excised specimens may appear slightly smaller than preoperative ultrasonographic measurements owing to partial decompression of the endometriotic content after excision.

Macroscopic appearance of excised specimens is shown in Figure 2.

### 3.5. Histopathological Findings

**Figure 3:**
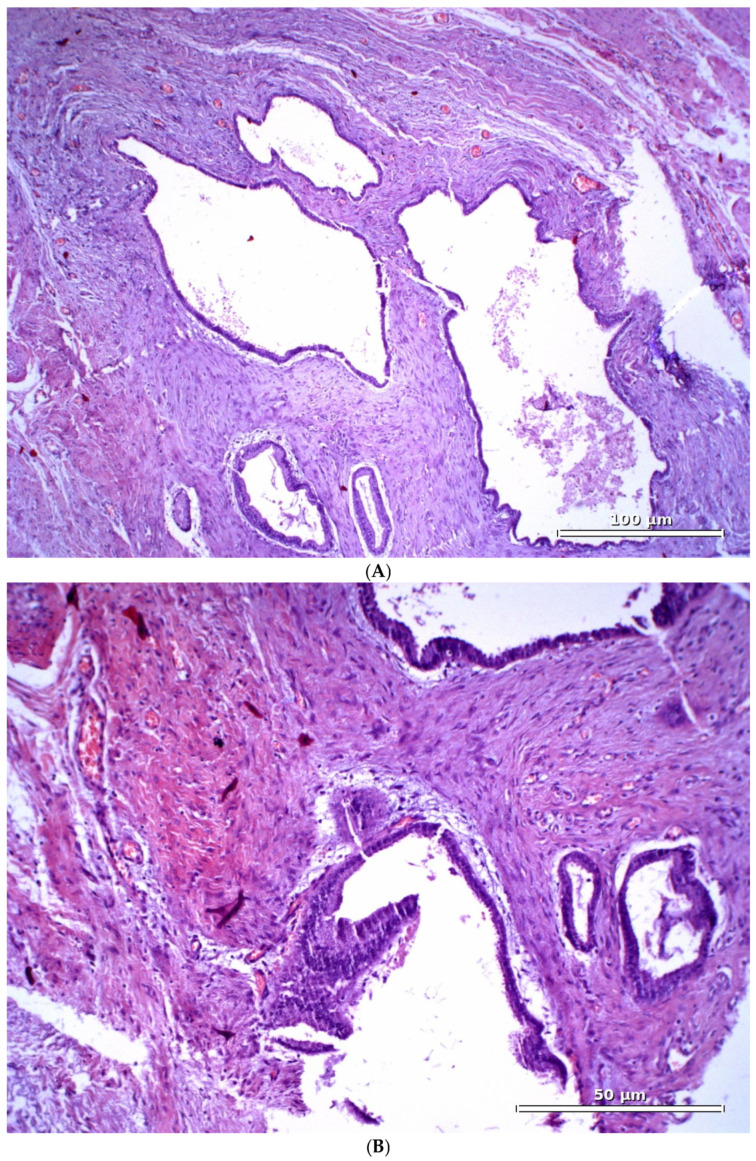
Representative histopathological photomicrographs (haematoxylin and eosin stain). (**A**) ×40 magnification (scale bar: 100 μm): endometrial glands and surrounding endometrial stroma embedded within fibrocollagenous tissue, consistent with endometriosis. Overlying epidermis and dermis are intact. (**B**) Shows ×100 magnification (scale bar: 50 μm): endometrial glands lined by pseudostratified columnar epithelium surrounded by cellular endometrial stroma, confirming the histopathological diagnosis of endometriosis.

All 14 specimens confirmed endometriosis: endometrial glands lined by pseudostratified columnar epithelium surrounded by endometrial stroma within fibrocollagenous tissue. Free surgical margins were confirmed in all cases. Representative photomicrographs are shown in Figure 3.

### 3.6. Postoperative Course and Follow-Up

One patient (Case 12) developed superficial wound discharge on postoperative day 5, which resolved completely with local wound care within two weeks, without antibiotic therapy or surgical re-intervention. No other wound complications were observed. Median hospital stay was 1 day (range 1–2) for subcutaneous cases and 2 days for intramuscular cases. Follow-up ranged from 12 to 24 months; three patients completed 24-month follow-up without evidence of recurrence. No recurrences or incisional hernias were identified in any patient at the time of last follow-up. Five patients (35.7%), comprising all three with intramuscular involvement and two subcutaneous cases with large lesions, were considered at higher risk for recurrence and were offered postoperative hormonal suppression; all five accepted and received dienogest (Visanne^®^ 2 mg/day). Information regarding preoperative hormonal therapy or counseling before referral was not systematically documented in the medical records and could not be analyzed.

## 4. Discussion

This retrospective case series of 14 patients with AWE following CS provides clinically relevant observations regarding intramuscular rectus abdominis involvement, preoperative imaging, primary fascial reconstruction, and postoperative recurrence. Although intramuscular invasion has been previously reported, published series specifically addressing its surgical management and fascial repair without prosthetic mesh remain limited [16,17,18]. Our findings complement the existing literature and support a systematic approach to operative planning in this uncommon presentation.

Intramuscular rectus abdominis involvement: Three of 14 patients (21.4%) had AWE with intramuscular invasion of the rectus abdominis muscle. This proportion is consistent with published series, in which intramuscular involvement has been reported in 9–30% of AWE cases [19,20,21]. Unlike superficial subcutaneous lesions, which can usually be excised without muscular reconstruction, intramuscular AWE requires deeper dissection, en bloc muscle resection, and primary muscular and fascial repair. The extent of muscular resection and the need for fascial reconstruction are directly related to the depth and size of the lesion: deeper lesions generally require wider resection margins and more complex repair, whereas smaller, superficially intramuscular nodules may be managed with more limited muscle excision. In all three of our intramuscular cases, fascial defects measured approximately 2 cm and were repaired primarily without prosthetic mesh, with no hernias at 12-month follow-up. These findings are consistent with the threshold of >2 cm proposed by Fernicola et al. [21] as the point at which mesh reinforcement may warrant consideration. Isolated rectus abdominis muscle endometriosis has been described as a distinct clinical entity with unique operative challenges [22]. Preoperative identification of the anatomical plane of involvement is critical for surgical planning; US was adequate for this distinction in all our cases, and MRI may be reserved for large or atypical lesions [13].

Scar location: Nodules were distributed along the right side (35.7%), left side (35.7%), and central portion (28.6%) of the Pfannenstiel incision, with a slight predominance of lateral locations. This distribution is consistent with the hypothesis that endometrial cell inoculation occurs preferentially at the lateral angles of the uterine incision during CS, where instrument and gauze exchange between the uterine cavity and the abdominal wall is most common [7,22].

Imaging: Ultrasonography was the primary imaging modality and was sufficient for lesion characterization and surgical planning in the majority of patients. Cross-sectional imaging (MRI or CT) may be considered for lesions with uncertain depth of involvement or diameters ≥35 mm, consistent with published recommendations [12,13]. In our series, this selective approach was applied in four patients with the largest lesions and proved adequate for preoperative mapping. The depth of invasion, as determined by preoperative imaging, directly influences the extent of muscular excision and the potential need for fascial reconstruction, and is therefore central to operative planning.

Clinical features and outcomes: The clinical profile of our patients is consistent with the established literature: women of reproductive age with prior CS presenting with cyclic painful masses at the Pfannenstiel scar and characteristic hypoechoic ultrasonographic appearance [4,10,12]. Free surgical margins and zero recurrences at 12 months were achieved in all 14 cases, consistent with larger published series [21,23]. A macroscopic excision margin of ≥1 cm is supported by our findings and endorsed in the literature [14,21]. Thirteen of 14 patients were managed without general anesthesia and were discharged within 24 h, reflecting the extraperitoneal and abdominal wall-confined nature of these lesions and supporting individualized anesthetic planning as a feasible and safe option in appropriately selected patients [24]. Postoperative hormonal suppression with dienogest was reserved for patients considered at higher risk for recurrence—specifically those with intramuscular involvement or large lesions—consistent with the rationale of reducing residual estrogenic stimulation following surgicalexcision [16,25].

Limitations: The retrospective design and single-center setting limit the generalizability of our findings. The sample size (*n* = 14) accumulated over approximately 11 years is consistent with the rarity of the condition but precludes inferential statistical analysis. The true institutional incidence of AWE may be underestimated: patients diagnosed at our center who subsequently underwent surgery elsewhere, and cases managed by the General Surgery department, were not captured in our records. Several clinically relevant variables—including the interval from cesarean section to symptom onset, body mass index, history of pelvic endometriosis, and preoperative hormonal counseling—could not be retrieved reliably. In particular, data on preoperative medical therapy or hormonal suppression offered before referral were not systematically documented, representing a meaningful limitation regarding counseling practice. The follow-up period of 12–24 months, while sufficient to detect early recurrence, may underestimate the true recurrence rate, as late recurrences beyond one year have been documented [16,25].

## 5. Conclusions

AWE following CS should be actively considered in any woman of reproductive age presenting with a painful or palpable mass at or near the Pfannenstiel scar. Preoperative ultrasonographic evaluation is the cornerstone of diagnosis; cross-sectional imaging may be reserved for cases with equivocal depth of involvement or large lesion size. Intramuscular rectus abdominis involvement, present in 21.4% of our series, requires careful operative planning including muscle resection and primary fascial repair; the extent of this repair is proportional to the depth and size of the lesion. Wide local excision with a margin of at least 1 cm and primary closure of fascial defects approximately 2 cm in size may be sufficient without prosthetic mesh augmentation. Postoperative hormonal suppression should be considered in patients at higher risk for recurrence. Individualized anesthetic planning, tailored to lesion depth and patient characteristics, should be integrated into surgical decision-making to optimize perioperative management. These findings should be interpreted as hypothesis-generating owing to the retrospective design and limited sample size. Larger prospective studies are required to define optimal surgical margins, recurrence predictors, and the role of adjuvant medical therapy in AWE.

## Figures and Tables

**Table 1:** Clinical and surgical characteristics of 14 patients with abdominal wall endometriosis following cesarean section.

Case	Age (y)	G/P	CS (n)	Plane	Symptom	Size (mm)	Site	Margins	F/U	Imaging
1	29	G2/P2	2	Subcutaneous	Cyclic pain + mass	20 × 25	Right	Free	NR	US
2	34	G3/P3	2	Subcutaneous	Cyclic pain + mass	20 × 15	Left	Free	NR	US
3	39	G2/P2	2	Subcutaneous	Cyclic pain + mass	21 × 20	Central	Free	NR	US
4	29	G3/P3	2	Subcutaneous	Cyclic pain + mass	23 × 23	Right	Free	NR	US
5	38	G3/P3	3	Subcutaneous	Cyclic pain + mass	15 × 15	Left	Free	NR	US
6	45	G3/P2	2	Intramuscular (rectus)	Cyclic pain	32 × 25	Right	Free	NR	US
7	32	G5/P2	1	Subcutaneous	Palpable mass	24 × 15	Central	Free	NR	US
8	45	G5/P3	3	Intramuscular (rectus)	Cyclic pain	11 × 15	Left	Free	NR	US + MRI
9	32	G2/P2	1	Subcutaneous	Palpable mass	16 × 9	Central	Free	NR	US
10	37	G3/P2	2	Subcutaneous	Cyclic pain	25 × 25	Right	Free	NR	US
11	31	G3/P3	2	Subcutaneous	Palpable mass	20 × 30	Left	Free	NR	US
12	45	G1/P1	1	Subcutaneous	Pain + mass	45 × 32	Right	Free	NR	US + CT
13	31	G1/P1	1	Intramuscular (rectus)	Pain + mass	27 × 30	Left	Free	NR	US + MRI
14	29	G2/P1	1	Subcutaneous	Palpable mass	21 × 11	Central	Free	NR	US + MRI

CS: cesarean section; G: gravidity; P: parity; F/U: follow-up; NR: no recurrence detected at 12-month follow-up; Scar site: anatomical location of the endometriotic nodule along the Pfannenstiel incision (right, left, central); MRI: magnetic resonance imaging; CT: computed tomography; US: ultrasonography.

## Data Availability

Data are available from the corresponding author upon reasonable request.

## Abbreviations

AWE	Abdominal wall endometriosis
CS	Cesarean section
CT	Computed tomography
MRI	Magnetic resonance imaging
US	Ultrasonography
WLE	Wide local excision
